# Initiatives to Support the Homelife of Women Physicians: A Systematic Review

**DOI:** 10.1007/s11606-025-09972-y

**Published:** 2025-11-19

**Authors:** Christina J. Kelly, Susanne Hempel, Diana Zhang, Sachi Yagyu, Aneesa Motala, Barbara J. Turner

**Affiliations:** 1 Keck School of Medicine, University of Southern California, Los Angeles, CA USA; 2 Department of Medicine, Keck School of Medicine of University of Southern California, Los Angeles, CA USA; 3 Department of Population and Public Health Sciences, University of Southern California, Los Angeles, CA USA; 4 Southern California Evidence Review Center, University of Southern California, Los Angeles, CA USA; 5 Annals of Internal Medicine, American College of Physicians, Philadelphia, PA USA; 6 Gehr Center for Health Systems Science and Innovation, Keck School of Medicine of USC, Clinical Sciences Center, Los Angeles, CA USA

**Keywords:** women physicians, homelife support, systematic review, breastfeeding support, maternity leave, gender equity

## Abstract

**Background:**

Women physicians are critical to healthcare delivery and have primary responsibility for homelife activities. Although diverse initiatives now support the work-related challenges of women physicians, published evidence about initiatives to address their homelife challenges has not been evaluated. We conducted a systematic review of interventions to mitigate diverse homelife responsibilities of women physicians.

**Methods:**

A comprehensive search was conducted for intervention studies with outcome measures related to childbearing, childcare, homelife responsibilities, and/or other family/personal support of women physicians in PubMed or PsycINFO and reference mining of publications. Publications at any time before September 15, 2025, were considered. Two independent reviewers screened studies and extracted data on the type of homelife initiative, study design, population, and setting for eligible studies. Results were extracted for prespecified outcomes: participation, uptake/response rates, effect on clinical outcomes, satisfaction, burnout, and practice engagement. Certainty of evidence and risk of bias of included studies were evaluated. No meta-analysis was performed due to study heterogeneity.

**Results:**

Of 2896 reviewed citations, 8 studies met inclusion criteria including 5 from the USA and 1 each from Turkey, Germany, and Japan. Sample sizes ranged from 6 to 790. Interventions with outcome measures directly affecting homelife addressed lactation support (4); pre- and post-partum leave (2); and assistance with housework (2). All studies were limited by non-randomized design and selection effects. Only the effects of lactation interventions were graded as moderately positive for breastfeeding duration and reduced interference with work. All studies had moderate or high risk of bias.

**Conclusion:**

The evidence base of interventions to assist women physicians with their homelife responsibilities primarily addressed childbearing and childcare. Studies were generally methodologically weak and failed to offer support to women who did not have children. This review identifies an important opportunity for research to support women physicians’ careers.

**Registration:**

CRD42025649041

**Supplementary Information:**

The online version contains supplementary material available at 10.1007/s11606-025-09972-y.

## INTRODUCTION

Concerns about physicians’ dissatisfaction with medical practice have been raised for over two decades.^1,2^ A narrative review of factors contributing to physician dissatisfaction in the early 2000 s reported that managed care, malpractice, health care inequities, and time pressures were among the most common reasons.^2^ Since that time, additional stressors have emerged, such as time-consuming electronic health record documentation and lack of control over the work environment,^3^ in the context of physicians increasingly becoming employees of large organizations.^4^ Compared with the general population, physicians have been reported to be more dissatisfied with work and more likely to burnout.^5^ Leaders in medicine have persuasively argued the business case for investing in physician well-being.^6^

Numerous initiatives have been described to improve physicians’ work environment such as promoting career advancement, offering mentorship, and developing a favorable organizational culture.^7–13^ However, women physicians also have primary responsibility for homelife activities, including childbearing, childcare, and other domestic roles such as preparing meals, housekeeping, and eldercare.^14,15^ Early career women physicians have been reported to delay childbearing, citing concerns about managing work with family demands and compromising career advancement.^16^ Despite efforts to improve the work environment, women physicians experience burnout more than male physicians ^17,18^ and have been more likely to leave clinical practice.^19^ Because women physicians serve an increasingly critical role in health care, both in the USA and abroad, a more comprehensive approach to supporting their careers would likely need to address their homelife responsibilities.

We conducted a systematic review of research publications on interventions implemented at work or outside of work but that were specifically designed to address women physicians’ homelife roles and responsibilities, ranging from childbearing support and childcare to help with domestic chores and family or personal care. This review aimed to answer the following question: “What is the effect of initiatives to support the homelife of women physicians?” To our knowledge, this is the first review to focus on approaches to support women physicians’ diverse homelife responsibilities. We offer insights into opportunities and significant gaps in evidence to support women physicians in managing demanding responsibilities at home while maintaining a demanding career in medicine.

## METHODS

This systematic review identified interventions that support women physicians’ homelife such as childbearing, childcare, domestic responsibilities, and eldercare. The search strategy for PubMed and PsycINFO combined terms for female physicians with potential homelife focus points such as ‘household work’, *childcare*, *eldercare*, *lactation*, *flexible scheduling *(e.g., “*flex time*,” “*job sharing*”), and *domestic supports *(e.g., “*meal preparation*,” “*concierge services*,” “*grocery shopping*,” “*domestic tasks*”) to identify empirical studies searched without date restrictions on September 12, 2025 (see Appendix). Reference mining of identified reviews, recommendations, and included studies complemented the search. We focused on female physicians because women often bear a disproportionate share of home responsibilities. The review is registered in PROSPERO and follows standard reporting guidelines.^20,21^

The review aimed to answer the following question: What are the effects of initiatives to improve homelife of women physicians? Two independent reviewers applied the following study eligibility criteria:

- Population: Women at all phases of physician medical training and/or completed training; practicing in academic or community-based healthcare settings and/or engaged in research in academic settings.
- Intervention: Interventions related to home activities of women physicians such as pre- or post-partum and lactation support; child rearing and childcare; maintaining a home; or other family-related responsibilities such as eldercare. We considered interventions focused on work-related roles such as flexible scheduling, but the study had to address an aspect of homelife. We excluded interventions that addressed work and career-related functions such as mentoring, career advancement support, work satisfaction, and management/leadership skills.
- Outcome: Studies that reported the effects of an intervention on the participants and directly related to homelife responsibilities such as childbearing, childcare, and/or other family/personal responsibilities. Excluded studies only described an intervention without structured data collection to assess effects on participating women.
- Design: Studies with a historic (pre-post, time series) or concurrent (e.g., controlled trials, randomized controlled trials) comparator that reported on the effect of an intervention. Excluded studies did not compare with another intervention, unknown status before the intervention, or treatment as usual. We also excluded studies that were only reported in abbreviated form (apart from trial records). Relevant reviews were not eligible for inclusion but were retained for reference mining.

Discrepancies in study eligibility assessments were discussed and resolved by consensus among the review team.

### Data Extraction and Quality Assessment

We extracted data on study setting, design, country, sample size, population characteristics (e.g., training level), and type of intervention. Outcomes were grouped into five domains: participation or uptake; satisfaction (e.g., wellness, institutional support); breastfeeding (e.g., duration, workplace accommodation); burnout and well-being; and career-related impacts (e.g., maternity leave, work status, or perceived career harm). When multiple measures or time points were reported for an outcome, we extracted the most complete or final result. Qualitative feedback relevant to each outcome was also summarized.

Data extraction was conducted by a content expert and independently reviewed by an experienced methodologist for accuracy and completeness. No automation tools were used, study investigators were not contacted for additional data, and no data conversions were performed. We relied solely on information reported in the publications and did not make assumptions or infer missing data.

Risk of bias and certainty of evidence were assessed by one reviewer and independently checked by a second. Discrepancies were resolved by discussion. Certainty was rated as high, moderate, low, very low, or insufficient based on predefined criteria: study limitations, imprecision, indirectness, inconsistency, and publication bias. Risk of bias was assessed using domains compatible with Cochrane RoB 2 and ROBINS-I (Appendix Table 1). Publication bias was not formally assessed due to the small number of included studies.

### Data Synthesis and Analysis

Effect measures, when reported, included proportions, odds ratios, and mean differences. Qualitative findings were summarized narratively. Given heterogeneity in study design, populations, and outcomes, we used narrative synthesis, reporting effect measures when available.

Two tables were developed from extracted data: one summarizing study characteristics, sample sizes, and effect measures; the other grouping findings by intervention type and outcome domain. No meta-analysis or formal sensitivity analyses were conducted. Prespecified subgroups included studies in academic medicine and non-US settings that targeted mainly trainee physicians.

### Role of the Funding Source

One author (C.K.) received support from the Gehr Center for Health Systems Science Summer Fellowship at the University of Southern California. The funding source had no role in the design, conduct, analysis, or reporting of the study, including the development or writing of the manuscript.

## RESULTS

**Figure 1:**
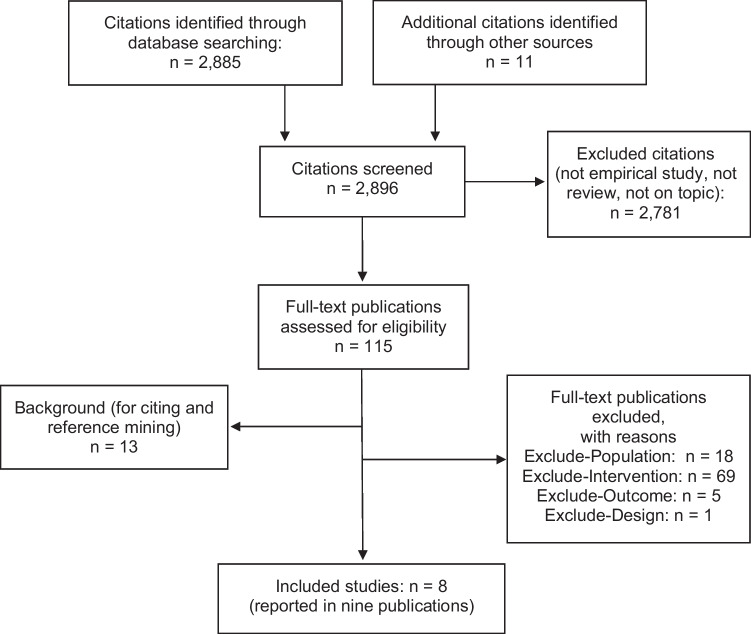
Literature flow diagram. Flowchart depicting the study selection process for the systematic review. A total of 2896 citations were screened, including 2885 from database searches and 11 from other sources. After exclusions, 115 full-text publications were assessed for eligibility. Of these, 8 studies reported in 9 publications were included in the final review. Reasons for full-text exclusions included ineligible population (*n* = 18), intervention (*n* = 69), outcome (*n* = 5), and design (*n* = 1). Thirteen additional studies were retained for background and reference mining only.

Of 2896 reviewed citations, eight studies in nine publications^22–30^ reported interventions that aimed to mitigate homelife responsibilities of women physicians (Fig. 1). Of 115 full-text publications assessed for eligibility, 93 were excluded for predefined criteria including the following: women physicians not studied, intervention unrelated to homelife responsibilities, and lack of data on structured outcome(s) or study design. Thirteen studies were identified as background papers. Full details are provided in Figure 1.

**Figure 2:**
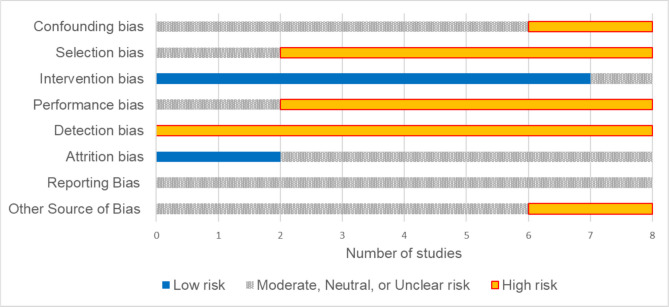
Risk of bias across studies. Bar chart showing the included studies (*n* = 8) judged for each bias type. Colors indicate risk ratings: blue = low risk, gray = moderate/neutral/unclear risk, orange = high risk. Bias types evaluated include confounding, selection, intervention, performance, detection, attrition, reporting, and other sources.

Five included studies were conducted in the USA and one in each of the following countries: Germany, Turkey, and Japan (Table 1). Six interventions offered pre- and/or post-partum support while two offered support for homelife responsibilities. None of the studies employed a rigorous design, such as a randomized controlled trial with random assignment to intervention groups. Risk of bias in the eight studies is summarized in Figure 2 (full rating in Appendix Table 1). Most studies introduced selection bias through their subject recruitment/selection; in addition, detection bias due to the data collection methods was a frequent limitation.

**Table 1:** Evidence Table: Interventions to Address Homelife of Women Physicians

Study ID	Population and design	Setting and intervention	Outcome and results
Atallah, 2024^30^ Dodelzon, 2021 ^24^	**Study population:** Radiology residents who were parents or expecting **Design:** Cohort **Total subjects:** *N* = 30 **Residents:** *N* = 4 **Practicing physicians:** *N* = 24 **Age:** NR	**Country:** USA **Setting:** Single academic radiology department **Homelife support focus:** Pre- and post-partum **Intervention description:** Parenting mentorship group; provision of wireless, wearable breast pumps	Participation in study Of 17 eligible trainees and faculty in the intervention period, 14 (82%) chose to use a wearable breast pump. The control group consisted of 13 participants who breastfed prior to the intervention Breastfeeding measures Satisfaction with lactational support was significantly higher in the intervention group (94%) compared to control (54%,) Continuation of breastfeeding upon return to work Use of the portable breast pump positively influenced decisions to continue breastfeeding after returning to work (median 4/5). Residents cited the convenience of wearable pumps as key to maintaining or extending breastfeeding Duration of breastfeeding 65% of participants rated the pump’s impact on breastfeeding duration as positive or very positive Parenting group evaluation 9/14 (64.3%) agreed that parenting groups were necessary for resident wellness Qualitative feedback Parenting group said to be “invaluable” and supportive Breast pumps reported to be convenient and easy to use at work
Colbenson, 2022^22^	**Study population:** Physicians and residents who have breastfed **Design:** Cohort **Total subjects:** *N* = 542 **Practicing physicians:** *N* = 329 **Residents:** *N* = 202 **Medical students**: *N* = 11 **Age range, y:** 18–25, 2 (0.3%); 26–30, 89 (16.4%); 31–35, 296 (54.6%); 36–40, 126 (23.2%); 41–45, 22 (4.1%); 46–50, 1 (0.2%); > 50, 1 (0.2%)	**Country:** USA **Setting:** Social media group **Homelife support focus:** Pre- and post-partum **Intervention description:** Provision of wireless, wearable breast pumps	Participants’ type of breast pump 321 (59.2%) subjects used a wearable pump and 221 (40.8%) subjects used a traditional pump Breastfeeding measures Able to breastfeed for intended duration 89.0% of wearable pump users versus 81.0% of traditional pump users (*p* = 0.005) Able to pump as often as needed at work 69.5% of wearable pump users versus 63.3% of traditional pump users (*p* = 0.16) Duration and frequency of breastfeeding Shorter breaks for lactation for wearable breast pump users versus traditional pump users (*p* < 0.0001) Frequency of breaks was similar (*p* = 0.22) Qualitative feedback on challenges to wearable breast pump use Concern about expense; No workplace policy or unsure of policy about wearing a pump in public
Creo 2018^23^	**Study population:** Pediatric residents recently returned from maternity leave and breastfeeding **Design:** Cohort **Total subjects:** *N* = 6 **Residents:** *N* = 6 **Age:** NR	**Country:** USA **Setting:** Single academic medical center **Homelife support focus:** Pre- and post-partum **Intervention description:** Provision of a dedicated lactation room with a hospital-grade breast pump for post-partum residents	Participation in study 6/7 (86%) of eligible residents participated Breastfeeding measures Pumping time reduced from a mean of 24 min to 15.5 min for portable pumps versus hospital pumps (95% CI: 3.8–12.2, *p* = 0.045) Milk volume per session increased from 6.0 oz to 8.8 oz for portable versus hospital pump (95% CI: 1.2–4.3, *p* = 0.06) Qualitative feedback Most residents reported greater comfort (5 of 6). All 6 reported less anxiety and guilt, improved engagement with clinical tasks and greater workflow integration for quieter hospital pump
Mourad, 2023^28^	**Study population:** Clinical faculty **Design:** Pre-post **Total subjects:** *N* = 70 baseline of whom 58 (83%) completed survey; 52 post-intervention **Practicing physicians:** *N* = 122 (respondent *N* = 106) **Age, y (mean ± SD):** 34.4 ± 2.4; 34.8 ± 2.7 post-intervention group	**Country:** USA **Setting:** Medical school **Homelife support focus:** Pre- and post-partum **Intervention description:** Increased availability of lactation rooms and relative value unit (RVU)-based compensation for lactation time	Participation in lactation support program survey Of 52 eligible faculty receiving intervention, 48 (92.3%) completed post-intervention survey Satisfaction measures pre- with post-intervention (five-point Likert scale) Finding time during clinic for pumping, mean 2.5 [SD 1.3] before versus 3.6 [SD 1.5] after intervention *p* < 0.001 Addressing the impact of lactation time on productivity (mean 2.0 [SD 1.0] versus 3.0 [SD 1.5], *p* < 0.001) Cultural support for lactation from colleagues and supervisors (mean 2.8 [SD 1.4] versus 3.4 [SD 1.3]; *p* = 0.047) Time reserved for lactation Mean 52.5 [SD 46.9] hours Reimbursement for RVU credits for time using lactation resources Among 40 subjects, total $242,744.37 dispensed (mean $6069/person)
Eren, 2018^25^	**Study population:** Women resident physicians delivering firstborn without complications before and after law **Design:** Pre-post **Total subjects:** *N* = 109 **Residents:** *N* = 109 Internal medicine or pediatrics (*N* = 66); surgery (*N* = 30), basic sciences (*N* = 13) **Age, y (mean ± SD):** 31.6 ± 4.18	**Country:** Turkey **Setting:** 3 hospitals in Istanbul **Homelife support focus:** Pre- and post-partum **Intervention description:** 2011 revision of Turkey’s maternity leave law including shortened work hours for 12 months post-partum, restricted night shifts, option to extend unpaid leave after standard 16-week paid leave	Participation in study 109/135 (80.7%) eligible residents participated 81 (74.3%) delivered before law and 28 (25.7%) after the law Maternity leave Uptake of maternity leave rose from 28.4% before to 50.0% afterward (OR = 2.74: 95% CI: 1.13–6.62, *p* = 0.037) Among internal medicine and pediatrics residents, duration of leave increased from mean 8.10 [SD 5.76] months to 9.50 [SD 3.12] months (*p* = 0.048) Breastfeeding Among pediatric and medicine residents, breastfeeding duration increased from mean of 12.39 [SD 6.67] months to 16.52 [SD 5.01] months (*p* = 0.016) No significant change in outcomes for surgical residents
Puhahn-Schmeiser, 2023^29^	**Study population:** Women medical students, residents, and physicians pregnant after amended Act **Design:** National cross-sectional survey **Total subjects:** *N* = 790 **Practicing physicians:** *N* = 163 **Residents:** *N* = 526 **Medical Students:** *N* = 83 **Others (not defined):** *N* = 18 **Age (mean ± SD), y:** NR	**Country:** Germany **Setting:** Medical Women’s Association members **Homelife support focus:** Pre- and post-partum **Intervention description:** 2018 amendment to Germany’s Maternity Protection Act to promote safer working conditions for pregnant physicians and medical students	Survey Participation 790 unique respondents Work-related effects 30% of respondents’ professional work hours reduced to 70% of pre-pregnancy hours Work restrictions were judged as reasonable (81.4%) 44.2% reported harming their career due to reassignment from clinical tasks or being excluded from training
Fassiotto, 2018^26^	**Study population:** Clinical and basic sciences faculty **Design:** Pre-post study **Total subjects:** *N* = 60 **Practicing physicians:** *N* = 60 **Age:** NR	**Country:** USA **Setting:** Single academic medical center **Homelife support focus:** Homelife responsibilities **Intervention description:** Integrated career-life planning and time-banking with credits to pay for academic or homelife support	Participation in survey Of 60 subjects, 42 (70%) completed the endpoint survey Impact of program (5-point Likert scale) Mean institutional satisfaction scores increased from 3.05 [SD 0.12] to 3.30 [SD 0.10], *p* = 0.020 Mean wellness scores increased from 1.89 [SD 0.12] to 2.13 [SD 0.13], *p* = 0.013 Mean support for culture of flexibility increased from 2.99 [SD 0.14] to 3.23 [0.12], *p* = 0.020
Fukuzaki, 2024^27^	**Study population:** Male and female physicians **Design:** Post-only **Total subjects:** *N* = 18 **Practicing physicians:** *N* = 18 (12 men, 6 women) **Age:** NR	**Country:** Japan **Setting:** Three academic medical centers **Homelife support focus:** Homelife responsibilities **Intervention description:** Household chore support services (1 or 3 visits)	Survey Responses (overall; women) Less burden by housework 14/18 (78%); 6/6 (100%) women. More family time 10/18 (56%); 3/6 (50%) women. More personal time 9/18 (50%); 3/6 (50%) women Qualitative feedback Favorable: 12/18 (6/6 women) participants cited lower stress, improved home cleanliness, and more time for personal/family activities Unfavorable: 6/18 participants concerned about: needing service more regularly; cost; scheduling constraints; and safety/privacy of cleaning person in the home

*CI* confidence interval, *N* number of participants, *NR* not reported, *SD* standard deviation

Four studies in the USA focused on facilitating and supporting lactation at work (Table 1). One pre-post intervention study offered professional hospital breast pumps and designated space for lactation to six breastfeeding pediatrics residents.^23^ The hospital machine significantly reduced the time to pump compared with these subjects’ prior use of a personal portable pump. An institutional program for radiology faculty and residents (reported in two publications) implemented a parenting program and provided wearable breast pumps.^24,30^ Participants endorsed the value of the program and the convenience of the wearable breast pumps. A survey of 542 practicing physicians or trainees in a social media group asked about the use of a wearable breast pump or a traditional lactation pump at work.^22^ Respondents who used the wearable pump were significantly more likely to report breastfeeding as long as they wanted, pumping as often as needed, and taking shorter work breaks to pump.^22^ Yet concerns were expressed about expense and the lack of workplace policies about the use of a wearable pump. Another intervention study for medical school faculty offered lactation resources at work with compensation for time reserved for lactation.^28^ Among 48 participants compared with 58 historical controls, satisfaction with lactation support on questions using a 5-point Likert-type scale was higher for finding time during clinic, maintaining productivity, and cultural support. Each participant received over $6000 in compensation for time spent using lactation resources.

Two international studies reported the effects of legal initiatives offering pre- and post-partum support. In Turkey, a law was passed to reduce work hours and night call, with the option for residents to extend their post-partum leave.^25^ At three hospitals in a study comparing outcomes after the law, the odds of taking maternity leave were nearly threefold greater for the 28 residents studied after the law versus 81 historical control residents. Benefits also included longer maternity leave and longer breastfeeding but only for internal medicine and pediatrics residents and not for surgical residents. In Germany, a maternity support law was amended to improve working conditions for pregnant physicians and trainees.^29^ Among 790 respondents, work hours were reduced for 30%. Although most respondents replied that these work limitations were reasonable, 44% were concerned about negative effects on their careers.

Two intervention studies addressed homelife support. A US academic medical center offered career planning and financial credits to faculty for extra work-related activities, including the option of using credits to pay for assistance with domestic responsibilities.^26^ Of 60 participating faculty, 42 (70%) responded to 5-point Likert-type questions before and after the intervention. Compared with baseline, a significant improvement was observed in mean wellness, satisfaction with the institution, and support for a culture of flexibility. Lastly, a Japanese academic medical center offered faculty one to three home visits by a service to support household chores.^27^ Of 18 participants, two-thirds reported favorable effects such as lower stress and more time for personal activities, but one-third expressed concerns about cost, safety, and privacy.

The summary of the design, findings. and certainty of evidence for three categories of interventions, including workplace lactation support (*n* = 4), national maternity leave laws (*n* = 2), and homelife support interventions (*n* = 2), showed that all were non-randomized and judged to be at high or moderate risk of bias (Table 2). Selection bias and reliance on self-reported outcomes were common. These limitations contributed to generally low certainty of evidence ratings. Only interventions to support lactation at work had positive effects, supported by moderate certainty of evidence regarding the longer duration of breastfeeding. Use of a wearable breast pump or a hospital-grade pump resulted in fewer interruptions at work. Satisfaction was good for the wearable breast pump but subjects often had concerns about cost and lack of institutional policies. The evidence was weaker for laws promoting maternity leave, which participants judged as acceptable but worried about negatively affecting their careers. Lastly, interventions to assist with home chores showed weak effects on satisfaction, wellness, stress, and perceived institutional support. None of the studies examined outcomes related to retention in work.

**Table 2:** Summary of Findings

Intervention focus (number of studies)	Outcome measures	Evidence	Reasons for downgrading	Grade
Lactation support (4 studies)^22–24,28,30^	Participation	2 studies reported high participation rates in pump programs: 82% for the wearable breast pump initiative and 86% for the hospital-grade pump program^23^	SL; IP	Very Low
	Uptake/ response rate	2 studies reported response rates that ranged widely from 58–92% 58% (14/24) residents in parenting mentorship and wearable breast pump intervention responded to post-intervention survey^24^ 92.3% (48/52) faculty in a lactation support intervention completed post-intervention survey^28^	SL	Low
	Effect on breastfeeding	4 studies indicated wearable pumps had positive effects on outcomes Breastfeeding for intended duration achieved more often for wearable pump users than traditional pump users (*p* = 0.005)^22^ Breaks for lactation shorter for wearable pump users than traditional pump users (*p* < 0.0001)^22^ Use of hospital-grade pumps decreased pumping time compared with portable pumps (*p* = 0.045)^23^ After a Turkish law promoting maternity leave, duration of breastfeeding increased for internal medicine and pediatrics residents (*p* = 0.016)^25^	SL; IC; IP	Very Low
	Satisfaction	3 studies reported increased satisfaction for wearable pumps and/or lactation space at work, but concerns also cited 1 study reported higher ratings for scheduling ease (*p* < 0.001), productivity (*p* < 0.001), and supervisor/colleague support (*p* = 0.047)^28^ 2 studies qualitatively reported that wearable pumps were more convenient than traditional pumps^22,24^ 1 qualitative study cited cost and unclear institutional policies as barriers to wearable pump use^22^	SL; IP	Low
Maternity leave laws (2 studies)^25,29^	Participation	1 study reported study participation 80.7% (109/135) of eligible residents participated in a study of maternity leave law^25^	IP	Low
	Uptake/response rate	No data	NS	Insufficient
	Effect on Maternity leave	2 studies reported longer maternity leave (pre- or post-partum) after laws Utilization of maternity leave rose from 28.4% before law to 50.0% afterward (OR = 2.74: 95% CI: 1.13–6.62, *p* = 0.037)^25^ Maternity leave increased from mean 8.1 months to 9.5 months for internal medicine/pediatrics (*p* = 0.048) (*p* = 0.037)^25^ 30% of respondents’ professional work hours reduced during pregnancy after law^29^	IP	Low
	Satisfaction	1 study reported respondents’ opinions about a law 81.4% of respondents rated work restrictions during pregnancy as reasonable^29^ 44.2% reported potential career harm from work restrictions^29^	NS	Insufficient
Support for homelife responsibilities (2 studies)^26,27^	Uptake/ response rate	2 studies reported survey response rates from 70 to 100% after interventions 70% (42/60) of physicians in a career-life planning intervention offering credits to pay for household chores responded^26^ 100% of 18 physicians receiving household-cleaning support responded^27^	SL; IP	Very Low
	Satisfaction	2 studies reported satisfaction or qualitative evaluation Mean scores increased significantly for satisfaction with the institution, wellness, and culture of flexibility^26^ All 6 women and 6/12 men valued lower housework burden, more family time, reduced stress, but had concerns about cost, scheduling, and safety/privacy when cleaners in the home^27^	SL; IP	Very Low
All interventions	Staying in medical practice	No data	NS	Insufficient
	Working full-time	No data	NS	Insufficient
	Burnout	No data	NS	Insufficient

*SL* study limitations, *IC* inconsistency, *IP* imprecision, *NS* no study, *NR* not reported, *SD* standard deviation, *CI* confidence interval, *OR* odds ratio

Certainty of evidence considered study limitations, inconsistency, indirectness, imprecision, and publication bias.

Final downgrading decisions were based on the following:

• Study limitations (SL): Downgraded when evidence was based exclusively on studies that had serious methodological limitations, including non-randomized, retrospective and/or self-selected populations, or unclear intervention classifications.

• Imprecision (IP): Downgraded when no effect estimates could be derived, wide confidence intervals were reported, or few events occurred that challenged the interpretation of the data.

• Indirectness (IC): Downgraded when evidence was exclusively based on a narrow clinical setting (e.g., single institution or specialty) that may not generalize to the broader population of women physicians.

• No studies (NS): We determined the evidence for the prespecified outcome of interest to be insufficient when no study had been identified that reported on the outcome.

We differentiated five grades of confidence in the evidence statements:

• High indicates that we are very confident that an effect estimate lies close to the true effect for a given outcome, as the body of evidence has few or no deficiencies. As such, we believe the findings are stable: i.e., further research is very unlikely to change confidence in the effect estimate.

• Moderate indicates that we are moderately confident that an effect estimate lies close to the true effect for a given outcome, as the body of evidence has some deficiencies. As such, we believe that the findings are likely to be stable, but further research may change confidence in the effect estimate and may even change the estimate.

• Low indicates that we have limited confidence that an effect estimate lies close to the true effect for a given outcome, as the body of evidence has major or numerous (or both) deficiencies. As such, we believe that additional evidence is needed before concluding either that the findings are stable or that the effect estimate lies close to the true effect.

• Very low indicates that we have very little confidence that an effect estimate lies close to the true effect for a given outcome, as the body of evidence has very major deficiencies. As such, the true effect is likely to be substantially different from the estimated effect; thus, any estimate of effect is very uncertain.

• Insufficient indicates that we were unable to derive any evidence statements due to the lack of research, limitations in the evidence base, or conflicting results.

## DISCUSSION

Support for the careers of women physicians has high public health significance, given their critical contributions to healthcare delivery in the USA and globally.^31^ This systematic review searched for studies of interventions implemented at work or outside of work, such as policies or laws, that specifically addressed aspects of homelife. Despite substantial research to address women physicians’ work-life challenges,^32^ this review identified only eight studies of interventions focusing on homelife responsibilities such as childbearing, childcare, and other domestic roles that were published before September 2025. These studies in the USA and three other countries offered low- or insufficient-certainty evidence reflecting limitations in design and generalizability. Thus, we identified a significant dearth of research to support women physicians in managing their disproportionately greater responsibilities for childbearing, childcare, domestic chores, and family support, among other homelife roles.^33^

Among these studies, we observed the most promising effects for interventions at work that were specifically designed to support the homelife responsibility of breastfeeding. Three US-based studies aimed to improve lactation by women physicians and trainees, including the provision of wearable pumps, parenting mentorship, increased access to lactation spaces, and salary support for time using work-based lactation resources.^22,24,28^ These interventions resulted in longer breastfeeding and fewer challenges with breast pumping during the workday. A Turkish law supporting maternity leave was also associated with a longer duration of breastfeeding. These interventions have significant value for the health and well-being of both mother and baby.^34^ In the USA, a variety of promising initiatives for practicing physicians are underway but research studies have not evaluated their outcomes. For example, the Universities of Michigan and Wisconsin have implemented programs to support breastfeeding surgeons, but outcomes have not been reported.^35^ Additionally, the Accreditation Council for Graduate Medical Education (ACGME) now requires residency and fellowship programs to offer accommodation at work for lactation by trainees^36^ but adoption has been unclear.^37^ We are also unaware of studies evaluating the implications for women physicians of the Pregnant Workers Fairness Act (Pub. L. 117–328) (PWFA) that was signed into law in 2023.^38^ The PWFA requires employers to offer “reasonable” time for a break at work and a private, secure space for expressing milk and expands protections for employees with limitations arising from pregnancy.^39^

Two studies in Turkey and Germany examined outcomes following laws to support maternity leave. The Turkish study reported a significantly longer leave to care for the infant following the institution of the law but only among internal medicine and pediatrics residents and not surgical residents.^25^ Surgical residents and attendings have been reported to experience challenges with childbearing and lactation in 44 countries.^40^ The German study examined outcomes after a 2018 amendment to its Maternity Protection Act to reduce trainees’ and practicing physicians’ unsafe work conditions and to limit work hours during pregnancy.^29^ Most respondents to a survey deemed the law as being acceptable but over 40% were concerned about compromising their careers.^29^ In the USA, the Family Medical Leave Act offers up to 12 weeks of leave from work after delivery, flexible hours, and increased safety, among other provisions.^41^ Outcomes specific to women physicians have not been studied; according to a national survey, women physicians reported shorter maternity leave than nonphysician professionals and both groups were dissatisfied with leave and compensation.^42^

We identified only two studies of interventions that addressed homelife responsibilities.^26,27^ These studies examined the provision of household services and offering credits for institutional work to be used for either academic or personal needs. Participants reported improvement in housework burden, personal time, and overall wellness but were concerned about the availability of services and safety issues. No studies specifically examined the effect of any intervention on burnout or continuing clinical practice.

Limitations of this systematic review need to be acknowledged. First, there is no agreed-upon terminology for the type of interventions we sought for this review, making literature searches challenging despite a librarian’s assistance. Second, we considered interventions at work that directly examined outcomes specific to homelife, such as breastfeeding or childcare. Workplace interventions that increased time off, salary, or general wellness were also considered but none examined homelife outcomes related to having children, housekeeping, meal preparation, or caregiving. We also examined part-time residency interventions but, again, none examined homelife outcomes.^43^ Admittedly, work-related interventions to increase efficiency or career satisfaction may reduce physician burnout^44^ and thereby benefit homelife functions; this remains to be addressed in research studies. Third, identified studies only examined short-term outcomes, but a medical society has proposed support for lactation at work to reduce attrition from the medical profession.^45^ Fourth, this review focused on women practicing or training in medicine, but future reviews should consider other clinicians such as women who are advanced healthcare practitioners and nurses also must balance work and homelife demands. Fifth, the identified interventions primarily focused on women of childbearing age, but older women physicians may still need support at home for domestic responsibilities and caregiving.

This novel systematic review addresses an important unmet need to promote women physicians’ careers through interventions that relieve homelife challenges. Our review has several strengths including a broader scope than any investigation of this topic, no restrictions on the search time frame, and assessment of the quality of the evidence. Ultimately, diverse approaches may be required to alleviate pressures on women physicians in order to manage a demanding profession along with childbearing, childrearing, and domestic chores. Such homelife interventions may offer complementary benefits to those offered by work-related interventions aiming to reduce burnout and disaffection from work.

## Supplementary Information

Below is the link to the electronic supplementary material.

ESM 1 (DOCX 55.2 KB)
