# Supplementary material for: Initiatives to Support the Homelife of Women Physicians: A Systematic Review
Source: J Gen Intern Med. 2025 Nov 19;41(3):819–30. doi: 10.1007/s11606-025-09972-y (PMC12960989; doi:10.1007/s11606-025-09972-y)
Supplement: Supplementary file 1 — (DOCX 55.2 KB) [file 11606_2025_9972_MOESM1_ESM.docx]

**Appendix**

PubMed

September 15, 2025

1 ((home[Title/Abstract] OR domestic[Title/Abstract]) AND "work efficiency"[Title/Abstract])

2 "flex time"[Title/Abstract] OR "job sharing"[Title/Abstract] OR "flexible work"[Title/Abstract]

3 "home life" OR "life career interface" OR "work life integration" OR "personal satisfaction" OR "family" OR "flexible work" OR "work arrangement" OR "work arrangements" OR "job sharing" OR "household work" OR "household tasks" OR "laundry service" OR "laundry services" OR "maternity leave" OR pregnancy OR "motherhood" OR "parental leave" OR "breastfeeding" OR lactation OR "child care" OR "childcare" OR creche OR "infant" OR "parturition" OR "family planning" OR "elder care" OR "meal preparation" OR "eldercare" OR "house work" OR "concierge service" OR "concierge services" OR "grocery" OR "shopping" OR "domestic tasks" OR "domestic responsibility" OR "domestic responsibilities" OR "parental responsibility" OR "parental responsibilities" OR "work-family" OR "work life"

4 "work-life balance"[MESH] OR "personal satisfaction"[MESH] OR "household work"[MESH] OR "work schedule tolerance"[MESH]

5 #1 OR #2 OR #3 OR #4

6 Physicians, women[MESH]

7 "female physician"[Title/Abstract] OR "female physicians"[Title/Abstract] OR "physician gender"[Title/Abstract]

9 "physician mother"[Title/Abstract] OR "physician mothers"[Title/Abstract] OR "woman physician"[Title/Abstract] OR "women physician"[Title/Abstract]

10 #6 OR #7 OR #9

11 clinicalstudy[Filter] OR clinicaltrial[Filter] OR observationalstudy[Filter] OR randomizedcontrolledtrial[Filter] OR evaluationstudy[Filter] OR systematicreview[Filter]

12 "randomized controlled trial"[Publication Type] OR "controlled clinical trial"[Publication Type]

13 "randomized controlled trial"[Publication Type] OR "controlled clinical trial"[Publication Type] OR "comparative study"[Publication Type]

14 "retrospective studies"[MeSH]

15 law[Title]

16 "randomized"[Title/Abstract] OR "randomised"[Title/Abstract] OR "randomization"[Title/Abstract] OR "randomisation"[Title/Abstract] OR "randomly"[Title/Abstract] OR "trial"[Title/Abstract] OR "clinical trial"[Publication Type] OR "clinical trial"[Title/Abstract] OR "clinical trials"[Title/Abstract] OR "comparative study"[Title/Abstract] OR "comparative studies"[Title/Abstract] OR "comparative effectiveness study"[Title/Abstract] OR "comparative effectiveness studies"[Title/Abstract] OR "prospective" OR "pre-post" OR "before-after"

17 #11 OR #12 OR #13 OR #14 OR #15 OR #16

18 #5 AND #10 AND #17

20 #5 AND #10 AND #17 from 1990 - 2025

23 #5 AND #22 AND #17

24 #5 AND #22 AND #17 from 1990 - 2025

25 Editorial[ptyp] OR Letter[pt] OR Case Reports[pt] OR Comment[pt] address[pt] OR "autobiography"[pt] OR "bibliography"[pt] OR "biography"[pt] OR "case report"[tw] OR "case reports"[tw] OR "comment on"[All Fields] OR congress[pt] OR "focus group" OR interview OR "key informant" OR "key informants" OR "dictionary"[pt] OR "directory"[pt] OR "festschrift"[pt] OR "historical article"[pt] OR lecture[pt] OR "legal case"[pt] OR "news"[pt] OR "newspaper article"[pt] OR "patient education handout"[pt] OR "periodical index"[pt]

26 #24 NOT #25

PsycINFO

((title: ("home life") OR title: ("life career interface") OR title: ("work life integration") OR title: ("personal satisfaction") OR title: ("family") OR title: ("flexible work") OR title: ("work arrangement") OR title: ("work arrangements") OR title: ("job sharing") OR title: ("household work") OR title: ("household tasks") OR title: ("laundry service") OR title: ("laundry services") OR title: ("maternity leave") OR title: (pregnancy) OR title: ("motherhood") OR title: ("parental leave") OR title: ("breastfeeding") OR title: (lactation) OR title: ("child care") OR title: ("childcare") OR title: (creche) OR title: ("infant") OR title: ("parturition") OR title: ("family planning") OR title: ("elder care") OR title: ("meal preparation") OR title: ("eldercare") OR title: ("house work") OR title: ("concierge service") OR title: ("concierge services") OR title: ("grocery") OR title: ("shopping") OR title: ("domestic tasks") OR title: ("domestic responsibility") OR title: ("domestic responsibilities") OR title: ("parental responsibility") OR title: ("parental responsibilities") OR title: ("work-family") OR title: ("work life")) OR (abstract: ("home life") OR abstract: ("life career interface") OR abstract: ("work life integration") OR abstract: ("personal satisfaction") OR abstract: ("family") OR abstract: ("flexible work") OR abstract: ("work arrangement") OR abstract: ("work arrangements") OR abstract: ("job sharing") OR abstract: ("household work") OR abstract: ("household tasks") OR abstract: ("laundry service") OR abstract: ("laundry services") OR abstract: ("maternity leave") OR abstract: (pregnancy) OR abstract: ("motherhood") OR abstract: ("parental leave") OR abstract: ("breastfeeding") OR abstract: (lactation) OR abstract: ("child care") OR abstract: ("childcare") OR abstract: (creche) OR abstract: ("infant") OR abstract: ("parturition") OR abstract: ("family planning") OR abstract: ("elder care") OR abstract: ("meal preparation") OR abstract: ("eldercare") OR abstract: ("house work") OR abstract: ("concierge service") OR abstract: ("concierge services") OR abstract: ("grocery") OR abstract: ("shopping") OR abstract: ("domestic tasks") OR abstract: ("domestic responsibility") OR abstract: ("domestic responsibilities") OR abstract: ("parental responsibility") OR abstract: ("parental responsibilities") OR abstract: ("work-family") OR abstract: ("work life")) OR (Keywords: ("home life") OR Keywords: ("life career interface") OR Keywords: ("work life integration") OR Keywords: ("personal satisfaction") OR Keywords: ("family") OR Keywords: ("flexible work") OR Keywords: ("work arrangement") OR Keywords: ("work arrangements") OR Keywords: ("job sharing") OR Keywords: ("household work") OR Keywords: ("household tasks") OR Keywords: ("laundry service") OR Keywords: ("laundry services") OR Keywords: ("maternity leave") OR Keywords: (pregnancy) OR Keywords: ("motherhood") OR Keywords: ("parental leave") OR Keywords: ("breastfeeding") OR Keywords: (lactation) OR Keywords: ("child care") OR Keywords: ("childcare") OR Keywords: (creche) OR Keywords: ("infant") OR Keywords: ("parturition") OR Keywords: ("family planning") OR Keywords: ("elder care") OR Keywords: ("meal preparation") OR Keywords: ("eldercare") OR Keywords: ("house work") OR Keywords: ("concierge service") OR Keywords: ("concierge services") OR Keywords: ("grocery") OR Keywords: ("shopping") OR Keywords: ("domestic tasks") OR Keywords: ("domestic responsibility") OR Keywords: ("domestic responsibilities") OR Keywords: ("parental responsibility") OR Keywords: ("parental responsibilities") OR Keywords: ("work-family") OR Keywords: ("work life")))

AND

((((Index Terms: (human females))) AND ((Index Terms: (physicians)))) OR (((title: ("female physician")) OR (title: ("female physicians")) OR (title: ("physician gender")) OR (title: ("physician mother")) OR (title: ("physician mothers")) OR (title: ("woman physician")) OR (title: ("women physician"))) OR ((abstract: ("physician mother")) OR (abstract: ("physician mothers")) OR (abstract: ("woman physician")) OR (abstract: ("women physician"))) OR ((Keywords: ("physician mother")) OR (Keywords: ("physician mothers")) OR (Keywords: ("woman physician")) OR (Keywords: ("women physician")))))

AND

((((title: ("clinical study")) OR (title: ("clinical trial")) OR (title: ("observational study")) OR (title: ("randomized controlled trial")) OR (title: ("evaluation study")) OR (title: ("systematic review")) OR (title: ("comparative study")) OR (title: ("comparative studies")) OR (title: ("comparative effectiveness study")) OR (title: ("comparative effectiveness studies")) OR (title: ("prospective")) OR (title: ("pre-post")) OR (title: ("before-after")) OR (title: ("law")) OR (title: ("retrospective studies")) OR (title: ("retrospective*"))) OR ((abstract: ("clinical study")) OR (abstract: ("clinical trial")) OR (abstract: ("observational study")) OR (abstract: ("randomized controlled trial")) OR (abstract: ("evaluation study")) OR (abstract: ("systematic review")) OR (abstract: ("comparative study")) OR (abstract: ("comparative studies")) OR (abstract: ("comparative effectiveness study")) OR (abstract: ("comparative effectiveness studies")) OR (abstract: ("prospective")) OR (abstract: ("pre-post")) OR (abstract: ("before-after")) OR (abstract: ("law")) OR (abstract: ("retrospective studies")) OR (abstract: ("retrospective*")))))

AND

Publication Type: Peer Reviewed Journal

**Appendix Table 1**

| **Author, year** | **Confounding bias** | **Selection bias** | **Intevention bias** | **Performance bias** | **Detection bias** | **Attrition bias** | **Reporting bias** | **Other bias** | **Overall RoB** |
| --- | --- | --- | --- | --- | --- | --- | --- | --- | --- |
| Atallah, 2024(1) | High risk  No randomization, no control for confounding | High risk  self selection, | Low risk  clear intervention classification | High risk  knowledge of intervention likely influenced responses | High risk  Self-reported outcomes, no blinding or objective assessment. | Moderate/Unclear risk | Moderate/Unclear risk | Moderate/Unclear risk | High risk  Moderate-High, no randomization, self-selection, and self reported outcomes |
| Colbenson, 2022(2) | Moderate/Unclear risk | High risk  convenience sample wit no random assignment | Low risk  clearly defined groups | High risk  Blinding not possible | High risk  Self-reported outcomes with no objective validation | Moderate/Unclear risk  not explicitly discussed, only incomplete surveys, not dropout | Moderate/Unclear risk | Moderate/Unclear risk | High risk  Moderate -High |
| Creo, 2018(3) | High risk  No control for external variables | High risk  Small, self-selected, non-randomized sample. | Low risk  Pump types were clearly defined and used as intended. | High risk  Participants knew which pump they were using and chose when to use each. | High risk | Low risk  No missing data or dropouts. | Moderate/Unclear risk | Moderate/Unclear risk  Single-site, small pilot with possible institutional influence. | High risk |
| Eren, 2018(4) | Moderate/Unclear risk | Moderate/Unclear risk | Low risk  clearly categorized as pre- or post-law, with no misclassification risk | High risk  Physicians knew the law changed, possibly biasing their self-reported responses. | High risk  All outcomes were self-reported | Moderate/Unclear risk | Moderate/Unclear risk | Moderate/Unclear risk | Moderate risk |
| Fassiotto, 2018(5) | Moderate/Unclear risk | High risk  Teams and individuals volunteered for the intervention | Moderate/Unclear risk | Moderate/Unclear risk | High risk  Wellness and satisfaction were self reported, participants were not blinded | Moderate/Unclear risk | Moderate/Unclear risk | Moderate/Unclear risk | High risk  Voluntary, single arm intervention with self reported outcomes |
| Fukuzaki, 2024(6) | Moderate/Unclear risk | High risk  No randomization | Low risk  no ambiguity in group assignment or concern for crossover | High risk  Knowledge of service for free would easily bias to encourage broader implementation | High risk  Self-reported outcomes | Low risk  All participants that had intervention completed survey | Moderate/Unclear risk | Moderate/Unclear risk | High risk |
| Mourad, 2023(7) | Moderate/Unclear risk  potential confounders could not be fully controlled for | Moderate/Unclear risk  unknown post intervention response rate; also not randomized | Low risk  Faculty were assigned to pre-intervention or post-intervention groups based on the timing of their childbearing leave with no crossover; well-defined intervention | High risk  No blinding and Self-reported satisfaction surveys | High risk  subjective outcomes and no blinded assessors | Moderate/Unclear risk  Unknown postintervention response rate = risk of nonresponse bias. | Moderate/Unclear risk  Potential for selective outcome reporting no protocol provided | High risk  External factors (COVID-19, policy changes) could bias results. | Moderate risk |
| Puhahn-Schmeiser, 2023(8) | Moderate/Unclear risk | High risk  self-selection could favor those with strong opinions or those negatively impacted. | Low risk  policy change was clearly defined | Moderate/Unclear risk | High risk  Those assessing the outcome were self-reported with no possibility of blinding | Moderate/Unclear risk | Moderate/Unclear risk | High risk  Potential recall bias due to self-reported outcomes, lack of a control group, and possible inconsistencies in policy implementation across different institutions. | High risk |

**Note:** Risk of bias was assessed using domains adapted from the Cochrane RoB 2 and ROBINS-I tools to capture key sources of potential bias. We assessed the following domains:

- **Confounding bias**: Whether key confounding variables were likely present and whether they were controlled for in the analysis.
- **Selection bias**: Whether the selection process of how participants included in intervention groups were selected or allocated to groups may have introduced systematic differences independent of the intervention.
- **Classification of interventions**: Whether the intervention status was correctly identified and maintained throughout the study.
- **Performance bias**: Whether deviations from intended interventions occurred or participants were aware of the intervention and potentially modified their behavior.
- **Detection bias**: Whether knowledge of intervention status influenced outcome assessment or if outcome ascertainment differed across intervention groups.
- **Attrition bias**: The extent of missing data, dropout rates, and whether rates differed between groups, potentially introducing bias.
- **Reporting bias**: Whether a pre-specified analysis plan existed, numerical results likely were selected on the basis of the results, or key outcomes were not reported or inadequately reported or could not have occurred in time yet.
- **Other sources of bias**: Any other methodological concerns not covered in the domains above, such as early stopping or protocol deviations.

Each domain was rated as:

- **Low risk**: Bias unlikely or well-controlled (e.g., adequate blinding, no missing data)
- **Moderate/Unclear risk**: Some concerns, insufficient information, or minor limitations; this was the default when information was insufficient
- **High risk**: Clear methodological flaws likely to bias results (e.g., confounding not controlled, selective reporting)

Full definitions and rating criteria were developed prior to data abstraction and applied consistently across studies.

**Appendix File References**

1. Atallah J, Shah S, Atzil E, Dodelzon K**.** Impact of a department-sponsored portable breast pump initiative at a single institution. Clinical Imaging. 2024;113.

2. Colbenson GA, Hoff OC, Olson EM, Ducharme-Smith A**.** The Impact of Wearable Breast Pumps on Physicians' Breastfeeding Experience and Success. Breastfeed Med. 2022;17(6):537-43.

3. Creo AL, Anderson HN, Homme JH**.** Productive Pumping: A Pilot Study to Help Postpartum Residents Increase Clinical Time. J Grad Med Educ. 2018;10(2):223-5.

4. Eren T, Kural B, Yetim A, Boran P, Gökçay G**.** Breastfeeding experiences of female physicians and the impact of the law change on breastfeeding. Turk Pediatri Ars. 2018;53(4):238-44.

5. Fassiotto M, Simard C, Sandborg C, Valantine H, Raymond J**.** An Integrated Career Coaching and Time-Banking System Promoting Flexibility, Wellness, and Success: A Pilot Program at Stanford University School of Medicine. Acad Med. 2018;93(6):881-7.

6. Fukuzaki T, Ooba S, Namba N, Yamada N**.** Changes in University Hospital Physicians' Work and Family Lives Following Outsourcing of Housework Cleaning Tasks. Yonago Acta Med. 2024;67(1):75-9.

7. Mourad M, Prasad P, Wick C, Sliwka D**.** Physician Satisfaction With Lactation Resources Following an Intervention to Improve Lactation Accommodations. JAMA Netw Open. 2023;6(8):e2327757.

8. Puhahn-Schmeiser B, Hennel EK, Gross C, Raestrup H, Bühren A, Mangler M**.** Female physician and pregnancy- effect of the amended German maternity protection act on female doctors' careers. Innov Surg Sci. 2023;8(1):23-8.
